# Real-Time Regulation of Beam-Based Feedback: Implementing an FPGA Solution for a Continuous Wave Linear Accelerator

**DOI:** 10.3390/s22166236

**Published:** 2022-08-19

**Authors:** Andrei Maalberg, Michael Kuntzsch, Eduard Petlenkov

**Affiliations:** 1 Helmholtz-Zentrum Dresden-Rossendorf, 01328 Dresden, Germany; 2 Department of Computer Systems, Tallinn University of Technology, 19086 Tallinn, Estonia

**Keywords:** regulation, beam-based feedback, FPGA, linear accelerator, continuous wave

## Abstract

Control applications targeting fast industrial processes rely on real-time feasible implementations. One of such applications is the stabilization of an electron bunch arrival time in the context of a linear accelerator. In the past, only the electric field accelerating the electron bunches was actively controlled in order to implicitly stabilize the accelerated electron beam. Nowadays, beam properties are specifically measured at a target position and then stabilized by a dedicated feedback loop acting on the accelerating structures. This dedicated loop is usually referred to as a beam-based feedback (BBF). Following this, the control system of the electron linear accelerator for beams with high brilliance and low emittance (ELBE) is planned to be upgraded by the BBF, and the problem of implementing a designed control algorithm becomes highly relevant. In this work, we propose a real-time feasible implementation of a high-order $H_{2}$ regulator based on a field-programmable gate array (FPGA). By presenting simulation and synthesis results made in hardware description language (HDL) VHDL, we show that the proposed digital solution is fast enough to cover the bunch repetition rates frequently used at ELBE, such as 100 kHz. Finally, we verify the implementation by using a dedicated FPGA testbench.

## 1. Introduction

**Figure 1:**
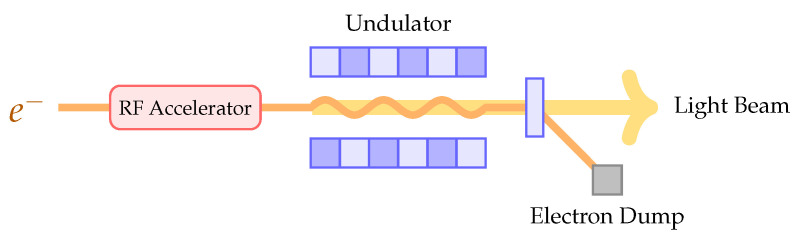
Conceptual schematic of an accelerator driven light source.

Radio frequency (RF) particle accelerators employ RF electromagnetic fields to accelerate charged particles to high energies while forming the particles into well-defined beams. A beam of accelerated particles can then be used to create a secondary radiation of ultra-short photon pulses, thus providing a light source for scientific experiments. Figure 1 illustrates a conceptual schematic of such a light source.

**Figure 2:**
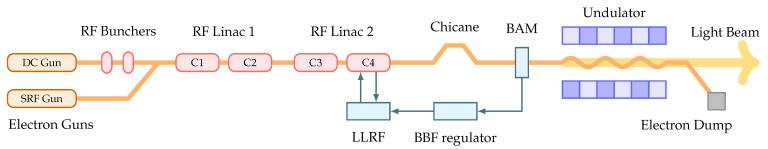
General layout of ELBE THz beamline featuring beam-based feedback regulation loop.

ELBE is a versatile light source located at Helmholtz-Zentrum Dresden-Rossendorf (HZDR), Germany. As illustrated in Figure 2, the general layout of the ELBE THz beamline follows the concept of a light source, namely: (1) electron bunches are generated by an electron gun, (2) these electron bunches are accelerated with the help of RF linacs consisting of superconducting RF cavities, and (3) photons are produced from these accelerated electron bunches inside a periodic magnetic structure, called undulator.

ELBE is one of the few electron linear accelerators routinely operated in a continuous wave (CW) mode. The notion of CW refers to a specific machine operation mode in which the RF electromagnetic field that resonates inside an accelerating RF cavity is driven continuously. Compared to a more common pulsed mode, CW allows flexible electron bunch repetition rates and high average current, thus enabling experiments that would otherwise be impossible to perform, hence the versatility.

Still, the quality of experimental results depends on the stability of the accelerated electron beam. For example, a time-resolved pump-probe experiment [1] may be configured to expect an electron bunch to arrive at the undulator with precise timing. In case this arrival time fluctuates due to the instability of the corresponding electron beam, this fluctuation is transferred to the subsequent secondary radiation, and the time resolution of such experiments degrades. Since the process of RF acceleration is affected by various disturbances, including RF noise inherent to RF acceleration modules and drifts caused by changes in ambient temperature, regulation of the electron beam becomes crucial.

The existing low-level RF (LLRF) controller installed at ELBE [2,3] represents the state of the art. The controller deals with stabilizing the RF field that accelerates the electron beam. However, such control scheme has no feedback from the electron beam, and thus there is no way to stabilize the electron beam directly. To overcome this limitation, the control scheme at ELBE is planned to be upgraded by a beam-based feedback (BBF) regulator, see Figure 2. This regulator is expected to minimize the impact of disturbances acting on the electron beam. In our previous work [4], we proposed a regulator design that seeks to minimize the fluctuations of beam arrival time with respect to low frequency RF noise. Due to CW mode, it was possible to target specific noise profiles, the low frequency ones included. This suggested the application of modern control techniques, such as optimal $H_{2}$ control [5,6], which finally resulted in a high-order state-space regulator. Even though the field of linear accelerators already lists a number of BBF control designs [7,8,9,10], these examples target pulsed machines. Yet ELBE is operated in CW mode, and very few studies [11] address real-time feasible implementations targeting CW machines. Especially, when the corresponding sample times are on a sub-microsecond level.

The bunch arrival time monitor (BAM) [12,13,14] measures the arrival time of every electron bunch accelerated inside the machine. This implies that the sample time of the regulator must correspond to the bunch repetition rate. Since this rate may reach several megahertz, the actual sample time requirement is forced to the sub-microsecond level. Obviously, such regulator requires an efficient, yet adjustable implementation of its algorithm, thus strongly suggesting an FPGA-based solution. In fact, low latency and high reconfigurability—the outstanding features of FPGAs—facilitated the adoption of FPGA-based solutions by the accelerator community [15,16,17,18]. As a result, the LLRF controller and BAM digital signal processing are implemented on FPGAs as well, thus establishing a low-latency digital signal path on the inputs and outputs of the regulator. It is therefore natural to follow the same trend and implement the regulator in terms of an FPGA-based solution.

In this work, we aim to fill the existing gap by presenting a real-time feasible FPGA-based implementation of the BBF regulator targeting the CW ELBE accelerator. We exploit the analogy between the state-space form of a digital system and a finite-state machine (FSM) to propose a general architecture of the digital solution. This architecture is then elaborated by introducing systolic arrays in order to deal with state-space matrix operations. Despite the fact that there are many sophisticated systolic array architectures [19,20,21,22], in this work, a straightforward systolic structure suffices that is similar to a 2-D array. Furthermore, we examine the application of fixed-point data types to the regulator algorithm. The latency of the resulting implementation is then compared to the one based on floating-point data types. Finally, by assembling a hardware testbench that involves another FPGA, we verify the output of our implementation against simulation data from Simulink.

The remainder of this paper is organized as follows: Section 2 presents the BBF loop structure at ELBE and discusses the loop elements, including the bunch arrival time regulator, the LLRF controller and the BAM sensor. The discussion aims to build a proper context for the regulator implementation. Section 3 describes the hardware and software frameworks used in this work. Following this, Section 4 introduces the resulting firmware architecture and demarcates the new regulator logic from the given framework. By moving towards internal details, Section 5 focuses on the implementation of state-space formalism and introduces important topics, such as systolic arrays and fixed-point data types. Section 6 demonstrates the hardware setup used to verify the regulator implementation and discusses the verification results. Finally, Section 7 concludes the paper.

## 2. Beam-Based Feedback Loop Structure at ELBE

**Figure 3:**
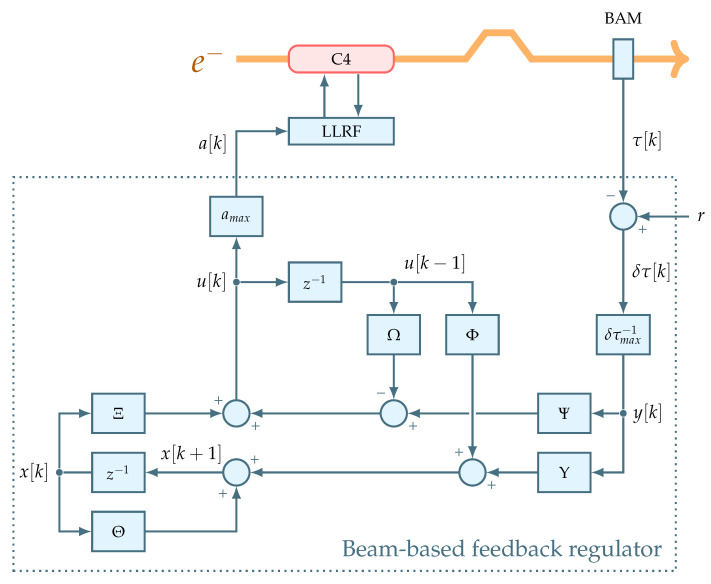
Beam-based feedback loop structure at ELBE.

ELBE beam-based feedback extends the existing control scheme by introducing a regulation loop involving a BAM sensor, a BBF regulator and a LLRF system of the fourth accelerating cavity (C4). This results in a new interconnection of control elements that involves cascaded loops. Figure 3 elaborates the emerging beam-based feedback structure, while making the main accent on the beam-based feedback regulator.

### 2.1. Beam-Based Feedback Regulator

The BBF regulator designed in [4] represents a 7th-order single-input single-output $H_{2}$ control law expressed in a state-space form

(1) $$\begin{array}{cc} x[k+1] & =Ax[k]+B_{2}u\left[k\right]+L_{x}e_{x}[k], \end{array}$$

(2) $$\begin{array}{cc} u[k] & =K_{u}x\left[k\right]+\;L_{u}e_{u}\left[k\right],\;\; \end{array}$$

where

(3) $$\begin{array}{cc} e_{x}\left[k\right] & =y\left[k\right]-C_{2}x\left[k\right]-D_{22}u\left[k\right], \end{array}$$

(4) $$\begin{array}{cc} e_{u}\left[k\right] & =y\left[k\right]-C_{2}x\left[k\right]-D_{22}u\left[k-1\right], \end{array}$$

where *x*, *u* and *y* denote the usual state, control input and measured output, and where *A*, $B_{2}$, $C_{2}$ and $D_{22}$ are the system, input, output and feed-forward matrices, respectively. Matrices $K_{u}$, $L_{x}$ and $L_{u}$ are the products of $H_{2}$ synthesis generated by MATLAB function h2syn. In order to alleviate the implementation of this control law, Equations (1) and (2) can be rearranged based on variables x[k], y[k] and u[k−1]. This yields a new set of matrices

(5) $$\begin{array}{cc} \Theta & =\;A+B_{2}K_{u}-B_{2}L_{u}C_{2}-L_{x}C_{2}-L_{x}D_{22}K_{u}+L_{x}D_{22}L_{u}C_{2}, \end{array}$$

(6) $$\begin{array}{cc} \Upsilon & =\;B_{2}L_{u}+L_{x}-L_{x}D_{22}L_{u}, \end{array}$$

(7) $$\begin{array}{cc} \Phi & =\;L_{x}D_{22}L_{u}D_{22}-B_{2}L_{u}D_{22}, \end{array}$$

(8) $$\begin{array}{cc} \Xi & =\;K_{u}-L_{u}C_{2}, \end{array}$$

(9) $$\begin{array}{cc} \Psi & =\;L_{u}, \end{array}$$

(10) $$\begin{array}{cc} \Omega & =\;L_{u}D_{22}, \end{array}$$

and since the new matrices can be evaluated offline, Equations (1) and (2) can be rewritten as

(11) $$\begin{array}{cc} x\left[k+1\right] & =\Theta x\left[k\right]+\Upsilon y\left[k\right]+\Phi u\left[k-1\right], \end{array}$$

(12) $$\begin{array}{cc} u\left[k\right] & =\Xi x\left[k\right]+\;\Psi y\left[k\right]\;-\;\Omega u\left[k-1\right]. \end{array}$$

As a result, Equations (11) and (12) form the structure of the beam-based feedback regulator displayed in Figure 3.

The regulator acts by correcting the setpoints of RF variables inside the LLRF controller FPGA. So to further reveal the loop details, the LLRF block displayed in Figure 3 needs to be expanded and elaborated.

### 2.2. RF System as Actuator for Electron Beam Regulation

**Figure 4:**
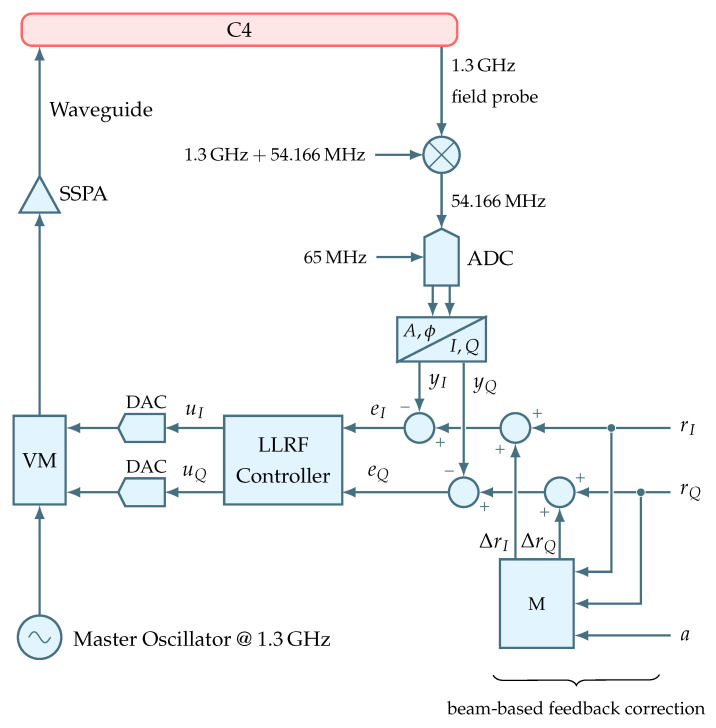
Schematic overview of RF system at ELBE including BBF extension.

In case of ELBE, every RF system is controlled by its dedicated LLRF controller. The purpose of such control is the stabilization of the corresponding RF variables, i.e., the amplitude *A* and phase ϕ of an accelerating RF field. Due to implementation specifics, the LLRF controller operates with the in-phase *I* and quadrature *Q* representations of the field amplitude and phase. Stabilized by the LLRF controller these *I* and *Q* signals are then applied inside a vector modulator (VM) to modulate a reference RF signal coming from a master oscillator. But since the signals generated by the LLRF controller are in the range of milliwatts, hence the name low-level RF, a solid-state power amplifier (SSPA) is used to drive the modulated RF signal to a kilowatt range. This finally results in a high-power RF signal that is passed through a waveguide to a RF cavity. Figure 4 illustrates the described RF system by expanding the LLRF block first introduced in Figure 3.

To incorporate the RF setpoint correction signal *a* coming from the beam-based feedback regulator, the LLRF controller FPGA is extended by a setpoint modulation logic *M*. The logic is defined as

(13) $$\left[\begin{array}{c} \Delta r_{I}\\ \Delta r_{Q} \end{array}\right]=\frac{1}{100}\left[\begin{array}{c} r_{I}\\ r_{Q} \end{array}\right]a,$$

where *a* denotes a change in the RF field amplitude expressed in percent units, and where $r_{I}$ and $r_{Q}$ are the RF field setpoints in *I* and *Q*, respectively. Factor 1/100 is required to convert the amplitude change in percent to a relative error ΔA/A [8]. In fact, this factor could be applied by the BBF regulator implementation. In this case, however, the small resulting number would suffer from underflows caused by the fixed-point data type in use by the LLRF implementation. Therefore, this factor is applied last. To sum up, the *I* and *Q* setpoints are modulated by the relative amplitude error in order to alter the RF field oscillating inside the cavity. This will cause a change in the arrival time of subsequent electron bunches, which can then be diagnosed by the BAM sensor.

### 2.3. Bunch Arrival Time Monitor as Sensor of Electron Beam

**Figure 5:**
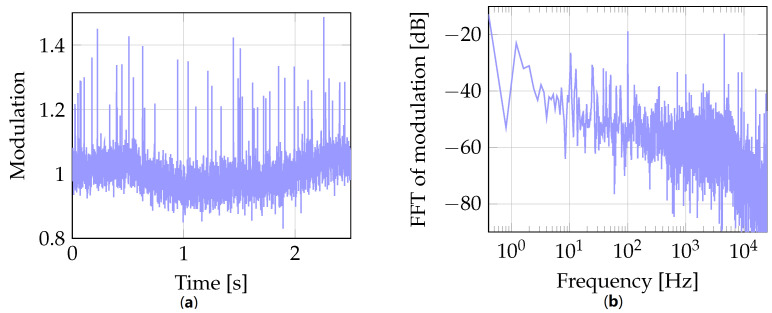
Example of BAM readout in (**a**) time and (**b**) frequency domains.

The beam-based feedback is represented by a bunch arrival time measured by the BAM sensor, see Figure 3. The operation principle of this sensor is based on measuring bunch arrival time relative to an actively-stabilized optical timing reference. Specifically, periodic pulses of a reference laser are amplitude-modulated with electric signals coming from pick-up antennas that probe the electric field of the passing electron bunch. The arrival time information is thus transferred into an amplitude modulation of coincident laser pulses. According to such arrival time representation, the output of this sensor is defined by a dimensionless modulation value τ with a working point set to τ=1. Figure 5 exemplifies a BAM readout taken at a bunch repetition rate of 50 kHz.

The BAM readout displays arrival time fluctuations caused by various disturbances, e.g., noise coming from the electron gun, drifts caused by changes in ambient temperature and RF noise inherent to RF acceleration process. In particular, the presented time domain data reveals distinct low frequency oscillations about the working point. Simultaneously, the frequency domain data features two components inherent to RF noise. These are (1) a random component represented by a spectral profile that decays with certain slopes as the frequency offset increases and (2) a deterministic component that manifests itself as a number of spurs along that profile. A harmonic of voltage ripple at 100 Hz is a representative of the latter.

**Figure 6:**
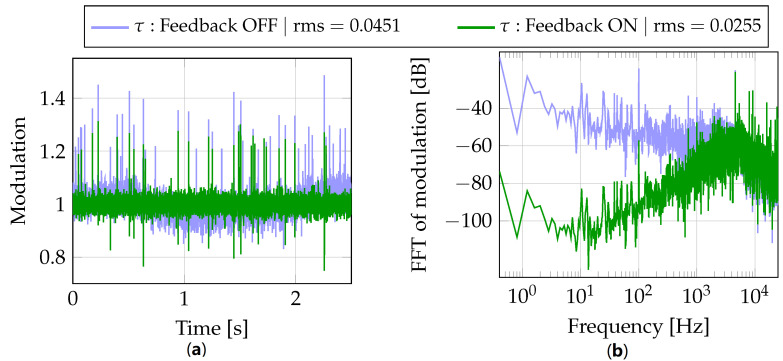
Simulation of BBF regulator with BAM readout in (**a**) time and (**b**) frequency domains.

The beam-based feedback regulator seeks to compensate the fluctuations in signal τ, and the simulation of such compensation is displayed in Figure 6.

The compensated data demonstrates the work of the regulator, i.e., (1) time domain data exhibits no drift and (2) the frequency data demonstrates suppression of low frequency noise up to ca. 5 kHz. Compared to the initial τ signal with no feedback, the regulator almost halves the disturbance effect in terms of the corresponding rms values.

The 50 kHz bunch repetition rate is commonly used for the ELBE THz beamline. Still, higher rates are possible, e.g., several hundred kilohertz or even a megahertz. Hence, the main reason to aim for a low-latency implementation of the regulator algorithm.

## 3. Hardware/Software Environment for Beam-Based Feedback Regulator

As a system, the beam-based feedback regulator relies on a number of subsystems to perform common tasks, such as establishing a user interface, sending and receiving of data, saving these data for further analysis, etc. To alleviate the heavy lifting, the regulator solution takes advantage of a digital platform based on the micro telecommunications computing architecture (MTCA) [23] and deployed on the ELBE accelerator machine [24].

### 3.1. MTCA.4 Hardware Environment

The fact that the regulator will be incorporated into the existing digital platform sets a few important conditions: (1) use of custom FPGA boards and (2) compliance with the existing firmware framework developed in VHDL [25]. Consequently, the regulator implementation is carried out on a specific data processing and telecommunication board, called the advanced mezzanine card (AMC) TCK7 [26]. The board features a high-performance FPGA device XC7K420T from Xilinx. To help leveraging the features of this board the given firmware framework provides support for

- RAM- or register-based internal interface (II) to communicate with CPU software;
- Low-latency links (LLLs) to communicate with other FPGA boards;
- Data acquisition (DAQ) facility to save diagnostic data to DDR3 memory.

**Figure 7:**
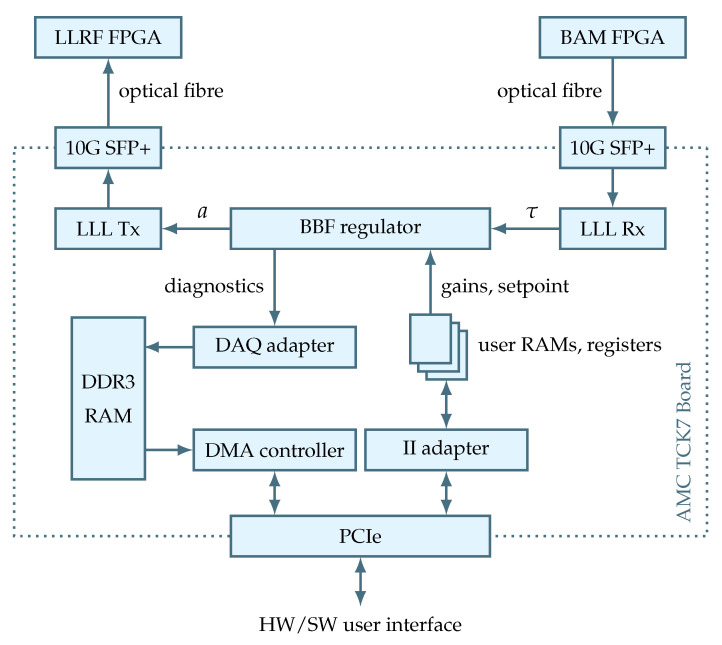
Beam-based feedback regulator in the context of MTCA.4 hardware environment.

In this work, the AMC TCK7 board must (1) receive data from the BAM FPGA, (2) do the processing and (3) send the result to the LLRF FPGA. While the processing is done inside the XC7K420T chip, the receiving/sending occurs through the low-latency links that are driven by 10-Gigabit small form-factor pluggable (SFP) optical transceivers. Measurement signals τ and control signals *a* are transferred via these LLL facilities. Similarly, the initialization of the processing stage happens through a register-based hardware/software (HW/SW) interface driven by a PCI Express (PCIe) connection. This interface is used by external CPU software to set the values of gain matrix elements, setpoints, etc. In addition, the processing stage needs to save diagnostics data. For this reason, DAQ facility is used to dump data to DDR3 memory. To sum up this description, Figure 7 illustrates a general block diagram showing the beam-based feedback regulator in the context of the given MTCA.4 hardware environment.

The complexity of the presented hardware system underscores the importance of a software-based user interaction. Hence, the necessity to establish a hardware/software interface that would facilitate the initialization, control and other user related tasks.

### 3.2. Hardware/Software Interface

**Figure 8:**
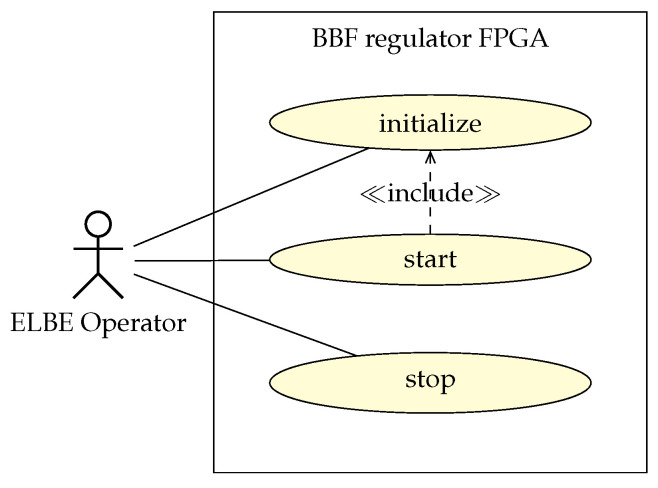
UML use case diagram summarizing user interaction with the BBF regulator FPGA.

The MTCA.4 technology uses a special software framework [27] to support the PCIe-based communication between the related hardware and software. Specifically, this framework provides the necessary application programming interface (API) to establish a register-based HW/SW interface. By writing and reading these registers the user is able to interact with the beam-based feedback regulator FPGA, and Figure 8 depicts a basic user interaction as a unified modeling language (UML) use case diagram.

**Figure 9:**
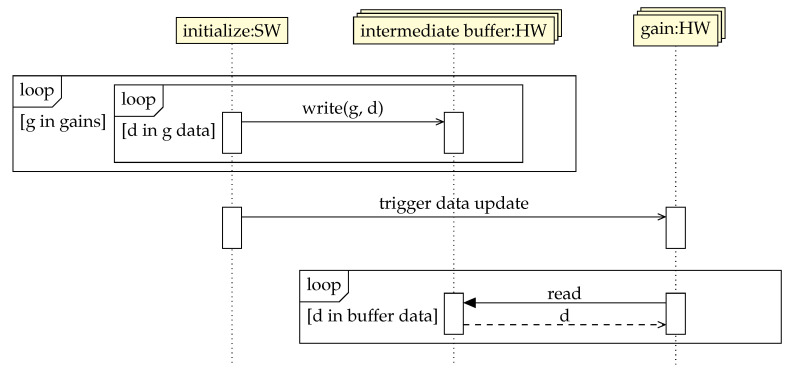
UML sequence diagram demonstrating the concept of (re)initialization of gain data.

Along with the basic use case to initialize the gain data when the application starts, a more sophisticated variant is to allow updating these data when the regulation is already running. The difficulty is related to the fact that ELBE is operated in CW mode, so there are no pauses long enough to permit a plain data overwrite. Moreover, it is reasonable to assume that updating the gain data separately, i.e., regardless of the algorithm state as a whole, will lead to erroneous algorithm results, hence the necessity to update the gains (1) all at once and (2) at a proper time instant. Essentially, the second requirement suggests an FSM-based implementation on the hardware side. In the meantime, the first requirement is fulfilled on the software side by first making a time-consuming write of the entire data from the software to intermediate hardware buffers and then triggering a fast data update on the hardware side. Figure 9 demonstrates this concept.

**Figure 10:**
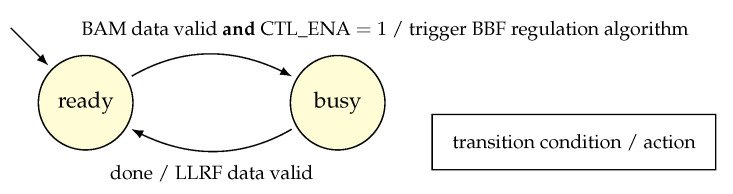
State diagram showing the effect of CTL_ENA flag on regulator state transitions.

The starting and stopping use cases are both based on the manipulation of the same flag to enable feedback data propagation to the regulation algorithm. This one-bit flag, called CTL_ENA, is set to 1 to enable regulation. Fundamentally, this defines an event-driven behavior of the algorithm implementation, i.e., when the algorithm receives no data, the regulation idles, see Figure 10.

In essence, the given MTCA.4 environment sets a number of requirements to the firmware architecture of the beam-based feedback regulator, namely:

- firmware is written in hardware description language VHDL;
- digital design targets an FPGA device from Xilinx;
- communication logic adheres to the given protocols, i.e., LLL and II;
- regulator algorithm is governed by an FSM.

With this in mind, the firmware architecture can now be elaborated.

## 4. Firmware Architecture of Beam-Based Feedback Regulator

**Figure 11:**
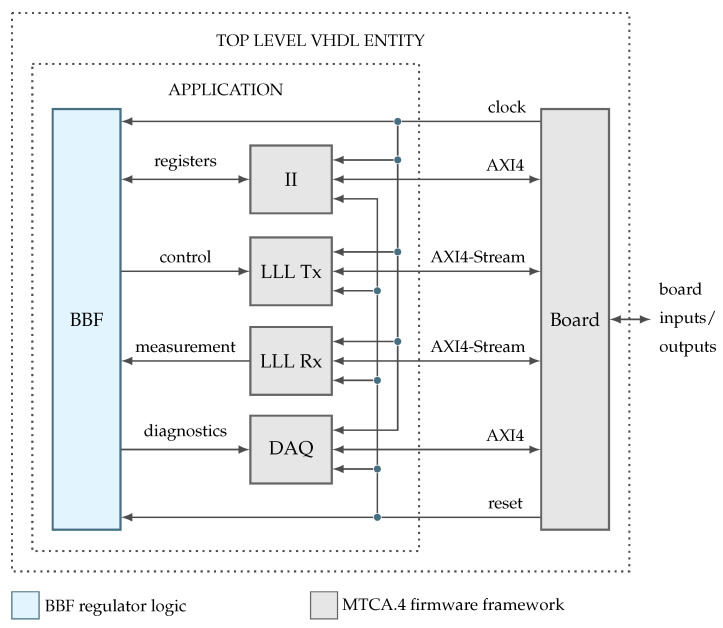
Structure of top level VHDL entity as defined by the MTCA.4 firmware framework.

The structure of the MTCA.4 firmware framework divides the on-chip logic into board and application compartments. The former manages the board specific features, including the low-level communication interfaces and clock generation, while the latter defines the application logic. Both compartments are then united inside a top level VHDL entity. Figure 11 depicts this structure as a general block diagram and uses color codes to demarcate the new regulator logic from the given framework.

### 4.1. Behavioral Model

The behavior of the regulator can be modeled in terms of the data that flows inside the application compartment and drives the initialization, regulation and diagnostics:

**Initialization**  Before running the regulation algorithm, the gain data need to be initialized. Since these data are transferred from the CPU software, the values are received by the firmware through the II communication. In fact, a single gain can be represented by a matrix with multiple values, so the receiver should be RAM-based. According to the firmware requirements outlined in Section 3, this RAM is expected to serve as an intermediate buffer that is first written by the software and then, after a software trigger, read by the actual gain logic. Once such initialization is complete, the regulation data flow becomes enabled.
**Regulation**  The regulation data flow starts from the reception of a BAM sensor measurement coming over a dedicated low-latency link. Provided the regulator algorithm is not busy at the moment and the regulation is enabled by the user, see Figure 10, the new measurement triggers the computation of a new control signal. Otherwise, the received measurement is dropped. Such behavior ensures that the algorithm always sees up-to-date measurements. When the algorithm is indeed triggered, the data starts flowing to gains and further to sums. Once the regulation algorithm is complete, the new control signal is transmitted over a dedicated low-latency link to the LLRF actuator.
**Diagnostics**  The diagnostics saves the regulation data, including the BAM sensor measurements and the corresponding control signals. Importantly, the BAM data are saved even if the regulator is disabled. This allows to diagnose the open-loop behavior of the system.

**Figure 12:**
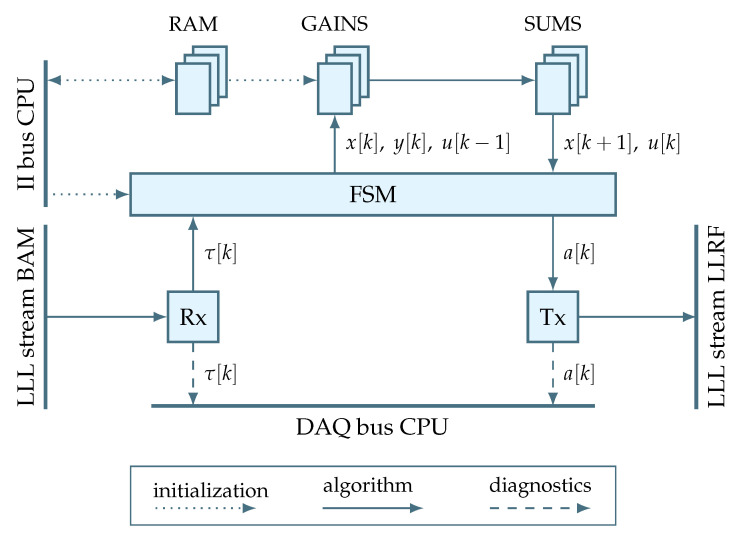
Functional schematic illustrating the main data flows inside the BBF application.

To sum up, Figure 12 illustrates a functional schematic of the BBF application. Note that the illustrated firmware blocks belong to the new regulator logic, hence the corresponding color code. In this context, the additional Rx and Tx blocks act as adapters between the framework and the regulator in order to establish a properly registered ready/valid handshake—a flow control technique [28] that is used throughout the entire regulator logic. Note also that for the sake of brevity the diagram omits the transformation from signal $\tau \left[k\right]$ to $y\left[k\right]$ and from $u\left[k\right]$ to $a\left[k\right]$. Finally, the diagram makes the central role of the FSM obvious.

### 4.2. Finite State Machine

**Figure 13:**
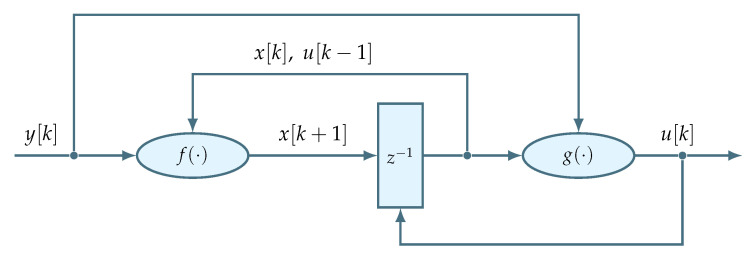
Discrete-time state-space regulator visualized as a Mealy FSM.

In addition to the firmware requirements outlined in Section 3, another reason for involving an FSM to manage the regulator algorithm is inspired by the analogy between a discrete-time state-space form of the regulator and a Mealy state machine [29]. Specifically, a hardware implementation of the Mealy machine is composed of combinational and sequential logic blocks. The combinational logic takes the current state together with input and computes the next state and output. The next state is then saved, or registered, by the sequential logic. The process repeats during the next iteration. Accordingly, Figure 13 depicts an FSM of the state-space regulator, where sequential logic block $z^{-1}$ registers the regulator state, and where combinational logic blocks $f\left(\;\cdot \;\right)$ and $g\left(\;\cdot \;\right)$ implement Expressions (11) and (12), respectively.

**Figure 14:**
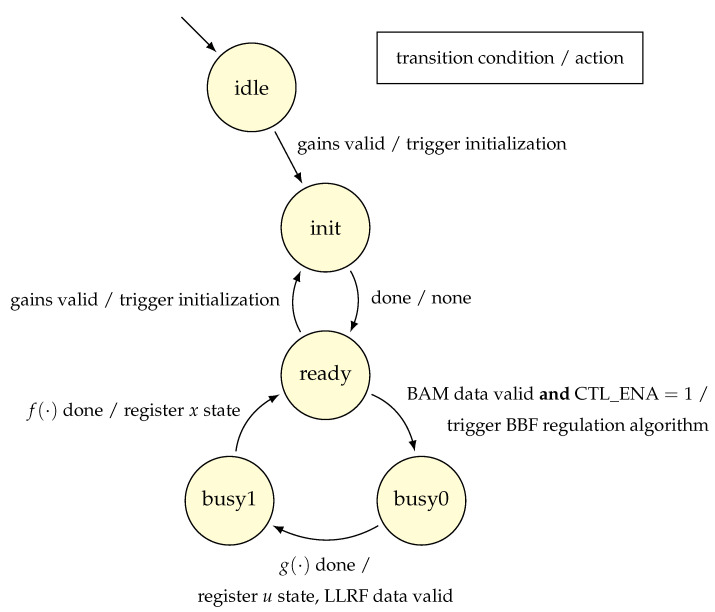
State diagram showing the operation of the regulator FSM.

Unfolding this FSM approach, Figure 14 demonstrates a state diagram that captures the essential parts of the regulator behavior. Note that once the BBF algorithm is triggered, $f\left(\;\cdot \;\right)$ and $g\left(\;\cdot \;\right)$ will be executed in parallel. Yet $f\left(\;\cdot \;\right)$ will take significant time to process its longest operation, i.e., Θx. So it is possible to get the result of $g\left(\;\cdot \;\right)$ and initiate the sending of LLRF data before $f\left(\;\cdot \;\right)$ completes. Hence, a busy state with two stages. Finally, the latency of algorithm operations, such as Θx, underscores the necessity to analyze the implementation of these state-space constructs.

## 5. State-Space Implementation in Hardware

In this work, we propose to describe the state-space formalism of the beam-based feedback regulator in terms of systolic arrays. Indeed, Equations (11) and (12) are based on matrix-vector multiplication, and systolic arrays offer a hardware architecture that enables massively parallel execution of this mathematical operation. Moreover, when implemented on FPGAs, systolic arrays can enjoy not only the inherently parallel nature of FPGAs, but also the availability of specialized circuits that are able to efficiently perform mathematical operations.

### 5.1. Systolic Array Structure

**Figure 15:**
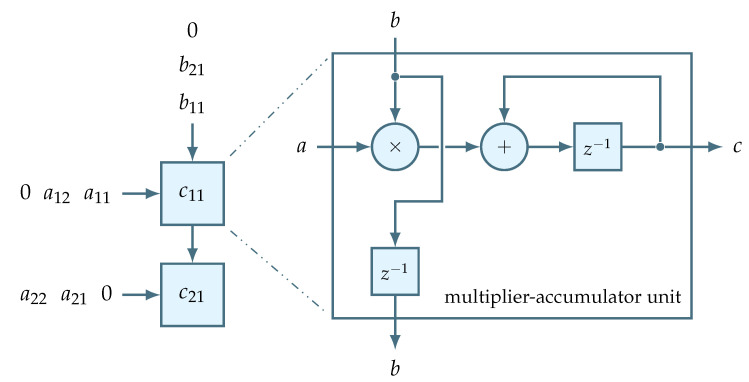
Structure of systolic array for multiplying a 2×1 vector b by a 2×2 matrix *A*.

Systolic arrays are grid-like interconnections of data processing elements, or nodes, that are driven by data to perform some specific uniform operation. In particular, a properly organized data flow can drive an array of multiply-accumulate nodes, or MACs, in order to implement a matrix-vector multiplication. Figure 15 demonstrates an example of a node interconnection that computes the product c=Ab, where *b* and *c* are 2×1 input and output vectors, respectively, and where *A* is a 2×2 matrix. In this straightforward systolic implementation the number of nodes corresponds to the size of the output vector *c*. This allows to parallelize the computation of individual output vector elements and to keep the computation results local to the nodes. Consequently, when the data finishes propagating through this interconnection in a wave-like manner, the nodes will store a fully computed output vector.

An efficient implementation of the presented systolic array node, i.e., the multiplier-accumulator unit, requires a design that exploits specialized computational resources provided by an FPGA, namely digital signal processor (DSP) slices. These high-speed circuits support a number of mathematical functions, including multiplication and addition, and therefore can accelerate a compute-intensive design. According to the data sheet [30], the XC7K420T device features 1680 DSP slices, and each slice contains a pre-adder, a 25 × 18 multiplier, an adder, and an accumulator. Correspondingly, this kind of slice can be configured to perform a multiply-accumulate operation in the form of a·b+c, where *a* and *b* are 25-bit and 18-bit signals, respectively, and where accumulation with *c* is sign-extended to 48 bits. On the one hand, by adhering to these signal widths, e.g., in case of custom fixed-point data types, a MAC unit can be mapped to a single DSP slice on the FPGA, thus leading to an optimal use of FPGA resources. On the other hand, if the MAC unit needs to operate with wider signals, e.g., when dealing with 32-bit floating-point data, the number of DSP slices per MAC unit increases, and the design consumes more FPGA resources. To investigate the optimal solution—both in terms of resources and latency—this work focuses on the custom fixed-point data types.

### 5.2. Fixed-Point Analysis of Regulator Signals and Gains

Due to the 25 × 18 signal widths dictated by the DSP48E1 slice [31], the fixed-point implementation differentiates between two types of data:

- signals flowing through the regulator, e.g., from $y\left[k\right]$ to $u\left[k\right]$;
- gain matrix values that modify the signals.

The signals and gains are assigned to 25-bit and 18-bit words, respectively, thus prioritizing the precision of the signal data. Apart from the precision, the 25-bit signal word needs to allocate enough bits for the integer part to avoid overflows inside the regulator. To mitigate this risk and to improve the conditioning of the regulator, the inputs and outputs are properly scaled. It is expected that the input signal coming from the BAM sensor exhibits a deviation of roughly $\delta \tau_{max}=0.1$. This yields $y=\delta \tau \;\cdot \;\delta \tau_{max}^{-1}=0.1\;\cdot \;10=1$, thus normalizing the signal magnitudes flowing inside the regulator.

However, there can also be unexpected spikes in the BAM readout, see Figure 5. In general, a large deviation of the input signal δτ will result in a considerable action taken by the regulator. In case of ELBE, a drastic change of the signal driving the cavity will have a high probability to trigger the ELBE protection system to switch off the accelerator. To cope with this unwanted scenario the signal δτ is limited as follows

(14) $$\begin{array}{cc} \delta \tau & =\left\{\begin{array}{cc} \delta \tau , & \mathrm{if}\delta \tau <\delta \tau_{lim}\\ \delta \tau_{lim}, & \mathrm{otherwise} \end{array}\right.. \end{array}$$

Setting $\delta \tau_{lim}=1$ yields $y=\delta \tau_{lim}\;\cdot \;\delta \tau_{max}^{-1}=1\;\cdot \;10=10$. Under these conditions, four bits are enough to represent the integer part of the signal. The derived signal data type is then (1, 25, 20), where the fixed-point notation is specified as

(15) $$\left(\mathrm{sign},\mathrm{word}\mathrm{length},\mathrm{word}\mathrm{fraction}\mathrm{length}\right).$$

In contrast, the fixed-point data type for gains of a 4th-order regulator is derived by examining the magnitudes of the values stored inside the corresponding matrices, namely

$$\begin{array}{cc} \Theta = & \left[\begin{array}{cccc} 47.0522 & -39.4131 & 1.48102 & -0.45106\\ 0.26835 & 99.3678 & 0.02415 & -0.00735\\ 13.2079 & 11.7310 & 99.0439 & 0.13750\\ -72.9060 & -102.943 & -7.72665 & 87.6644 \end{array}\right]\times 10^{-2},\\ \Xi = & \;\left[\begin{array}{cccc} -291.624 & -411.771 & -30.9066 & \;\;9.41304 \end{array}\;\;\right]\times 10^{-2}, \end{array}$$

$$\begin{array}{cccccc} \Upsilon = & \left[\begin{array}{c} -7.13823\\ -0.11638\\ -47.8240\\ 12.2410 \end{array}\right]\times 10^{-2}, & \;\;\;\; & \;\;\;\; & \;\;\;\;\;\;\Phi = & \left[\begin{array}{c} -2.34632\\ -0.03825\\ 0.71523\\ 12.2410 \end{array}\right]\times 10^{-2},\\ \Psi = & \;\;48.9641\times 10^{-2}, & & & \Omega = & -48.9641\times 10^{-2}. \end{array}$$

Depending on the magnitude range of a particular matrix, the gain data type can vary its precision. For example, the maximum magnitude among the matrix values constituting the gain Θ is −1.0294. Hence, only one bit suffices for the integer part, another bit represents the sign, and 16 bits can be allocated to the fraction part, thus yielding a precision of $2^{-16}=1.5259\times 10^{-5}$. Following this, Table 1 displays the various fixed-point data types assigned to the regulator matrices.

The gains $\delta \tau_{max}^{-1}$ and $a_{max}$ are integers with values 10 and 1, respectively, so their fraction parts could be omitted. Still, it is expected that these gains can be manipulated during run-time to do fine-tuning of the regulator. Thus, the fraction parts are preserved and the corresponding data types are both chosen as (1, 18, 10). In addition, the BAM and LLRF signals have fixed-point data types (0, 18, 15) and (1, 18, 10), respectively. Consequently, the regulator implementation needs to take this into account when receiving or sending low-latency link data.

The presented analysis underscores the fact that a fixed-point implementation requires a thorough examination of the data flowing inside the design. It is therefore of interest to see how much this effort helps to keep the systolic array structure optimal in terms of latency and resources, e.g., compared to a floating-point implementation.

### 5.3. Data Flow to Drive Systolic Arrays

**Figure 16:**
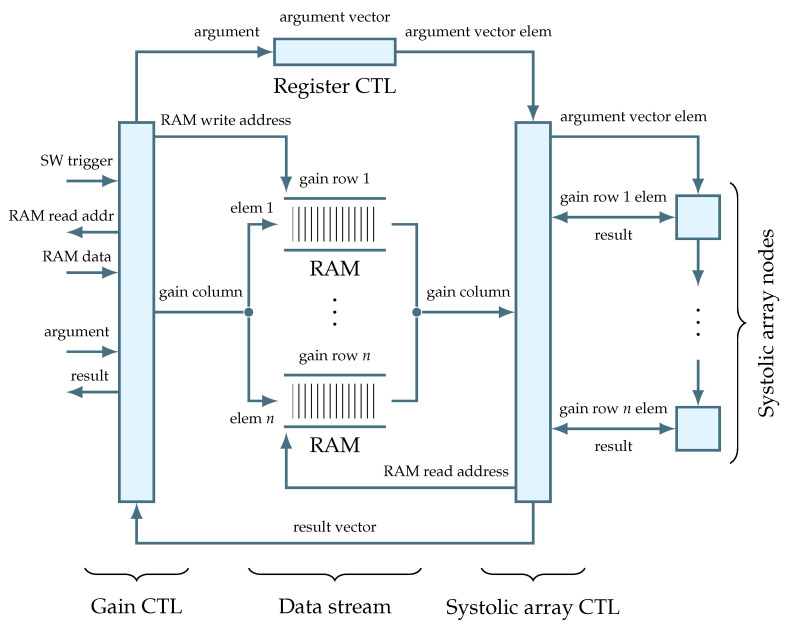
Gain entity schematic showing internal data flow.

The operation of systolic arrays relies on a properly organized data flow. In this work, a digital circuit responsible for the data flow is called a data channel, and multiple data channels compose a data stream. As shown in Figure 16, this circuit plays a central role in the gain entity.

Inside the data stream circuit, the data are structured based on their types. The gain is treated as a two-dimensional matrix and is organized into matrix rows. These rows are represented by separate data channels implemented as RAMs. Such organization facilitates the gain data throughput, because it allows to write or read an entire matrix column in a single clock cycle. This also places a requirement on the software to write the gain data to intermediate RAM buffers in a column-major order. After a software trigger, the gain controller will rely on the proper format of the intermediate data in order to initialize the internal data channels column by column. Unlike the gain, the signal is placed into a register-based memory. Again, this allows to register an incoming signal, i.e., the gain argument, in a single clock cycle. Along with the gain column, the signal vector element is then fed into the systolic array to perform the necessary computation. Once the computation is over, the results from each systolic array node are assembled into a result vector which is then propagated to the gain entity output.

**Figure 17:**
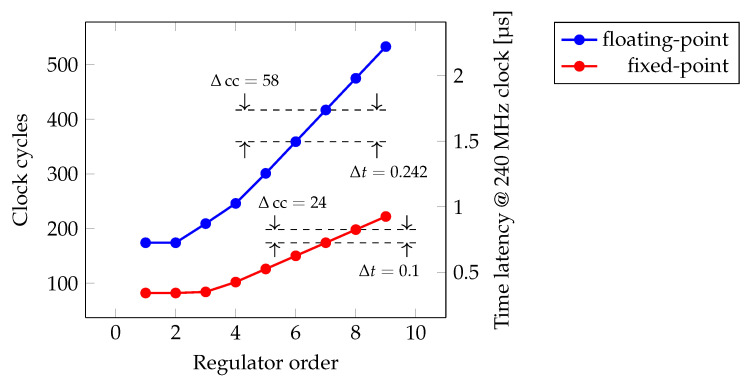
Time latency of the regulator busy state under various orders and data types.

Undoubtedly, the data stream circuit introduces some amount of overhead into the design. Moreover, the latency of the data management will dominate in case of low-order regulator implementations, see Figure 17.

The data stream overhead is partially related to data padding required by systolic arrays. As can be seen in Figure 15, the data flow driving the systolic array is padded with zeros. Although these zeros ensure the correct computation inside the nodes, they also lead to the increase of the corresponding data channel size which becomes

(16) datachannelsize=gainrowsize+gaincolumnsize−1.

Of course, the overall latency of the regulation algorithm is also affected by the implementation of the state-space computation units, i.e., the MAC and the sum. This is especially true for the floating-point case which uses a 32-bit single precision data type [32] and thus relies on an intellectual property (IP) from Xilinx [33]. In this context, even though the sum implementation represents a trivial element-wise summation of the argument vectors, and thus does not involve systolic arrays, its floating-point implementation still uses DSP resources, see Table 2.

Indeed, by adhering to the dictated signal widths the fixed-point MAC implementation is mapped to a single DSP slice. Compared to the floating-point counterpart that uses more DSPs per MAC, the one-to-one mapping allows the fixed-point design to occupy considerably less DSP resources on the FPGA.

It can be argued, though, that the usage of the proposed gain entity architecture for scalar operations, such as $\delta \tau_{max}^{-1}\;\cdot \;\delta \tau$, is far beyond what is required. Hence, the unwanted overhead. This is a reasonable point provided the system does not change in the future. Yet there are plans to extend the system in order to regulate the compression and energy of the electron bunches. In this case, the current architecture can be easily scaled to implement the required mathematical operation, e.g.,

(17) $$\left[\begin{array}{c} y_{\tau }\\ y_{E}\\ y_{C} \end{array}\right]=\left[\begin{array}{ccc} \delta \tau_{max}^{-1} & 0 & 0\\ 0 & \delta E_{max}^{-1} & 0\\ 0 & 0 & \delta C_{max}^{-1} \end{array}\right]\;\cdot \;\left[\begin{array}{c} \delta \tau \\ \delta E\\ \delta C \end{array}\right],$$

where *E* and *C* denote the energy and compression of the electron bunches, respectively. This underscores the scalability of the current digital solution.

In general, the presented data demonstrates that the proposed state-space architecture can be routinely clocked at 240 MHz—a particular clock signal generated by the board compartment of the MTCA.4 firmware framework. Running at such frequency allows the fixed-point implementation to stay on the sub-microsecond level even in the case of high-order regulator designs. The floating-point implementation exhibits greater latency, but even the 7th-order regulator designed in [4] takes less than 2 microseconds to perform its state-space computation. The 50 kHz bunch repetition rate is thus supported by both implementations. In the mean time, the fast fixed-point implementation needs to be verified in hardware.

## 6. Firmware Verification

**Figure 18:**
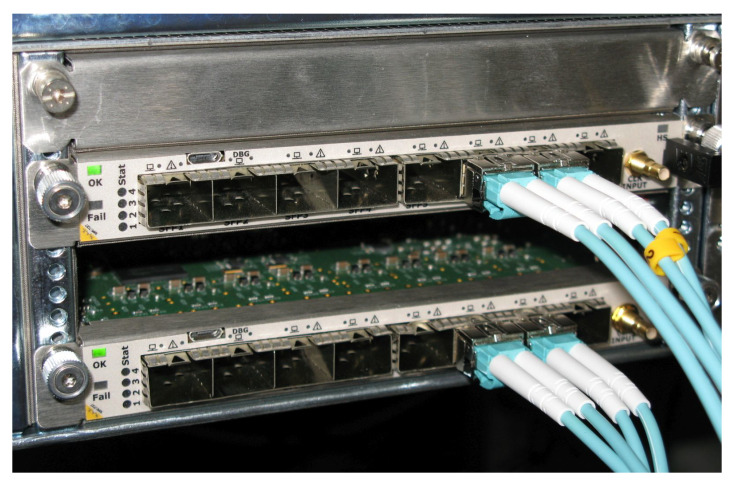
MTCA.4 crate with two installed FPGAs serving as a testbench.

The correctness of the regulator implementation is verified by assembling a digital setup which involves the BBF regulator FPGA connected to an additional FPGA that serves as a testbench. Along with the verification of the state-space implementation, the usage of a separate FPGA allows testing the low-latency link communication. The corresponding installation can be observed in Figure 18. The two FPGAs are slided into a MTCA.4 crate leaving only their front panels exposed. As can be seen, these panels feature SFP slots that are used to interconnect the two devices using optical cables.

In principle, the testbench operation can be outlined as follows

1. Sending a stimulus to the BBF regulator FPGA;
2. Receiving a response;
3. Comparing the received response with a similar one generated by a floating-point MATLAB simulation.

**Figure 19:**
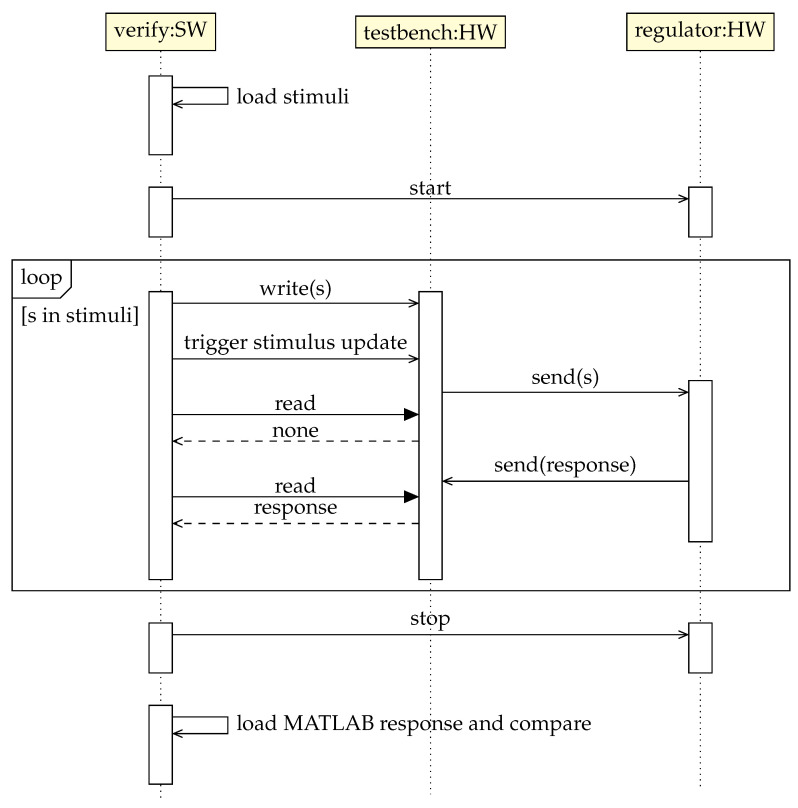
UML sequence diagram showing the operation of the BBF regulator testbench.

The MATLAB simulation is run offline and is driven by the same stimulus—simulated BAM data. Hence, both responses should be identical to a certain degree of precision. Figure 19 summarizes the outlined testbench operation.

**Figure 20:**
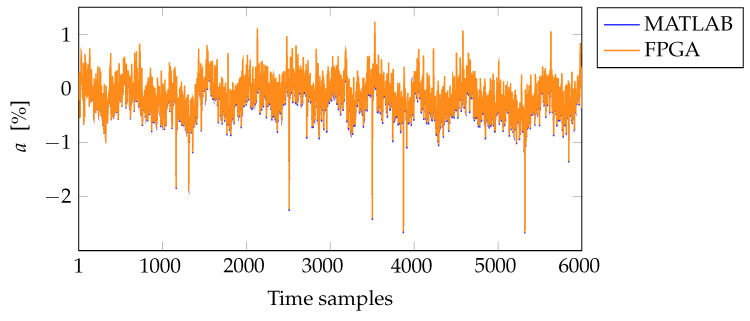
Comparison of MATLAB and testbench time responses for a 4th-order regulator.

In this work, the testbench compares the time response of a 4th-order regulator implemented in fixed-point with the same regulator simulated in a floating-point MATLAB model. The result of this comparison is depicted in Figure 20.

**Figure 21:**
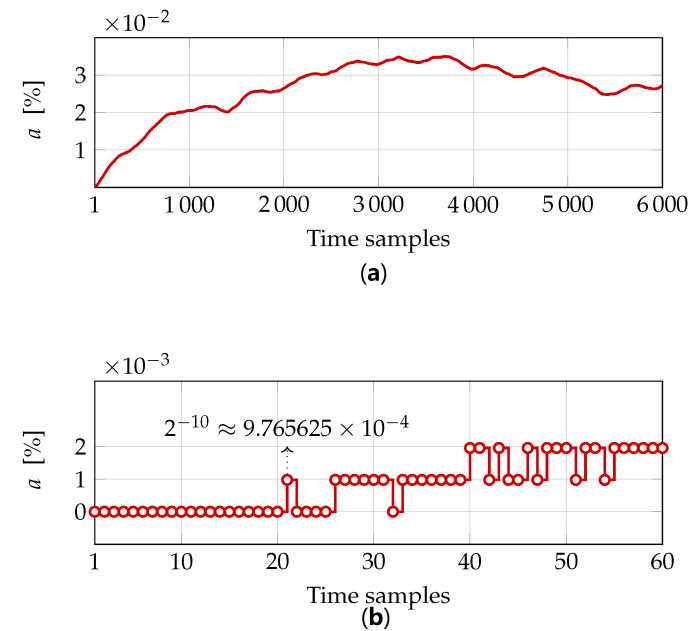
FPGA and MATLAB responses expressed as (**a**) absolute difference and (**b**) zoomed.

The difference between the response simulated in MATLAB and the one produced by the testbench is displayed in Figure 21. Essentially, the assembled testbench represents an open-loop system, i.e., the output of the testbench regulator has no effect on the next BAM data sample. Coupled with the precision issue, the open-loop scenario leads to accumulation of the mismatch between the two responses. Still, it is expected that this negative behavior will disappear, once the fixed-point regulator closes the real machine loop. In the closed-loop scenario, the correctness of the first few responses will matter, and the current testbench data exhibits up to 20 correct responses before the least significant fraction bit flips. Note that the magnitude of the bit flip corresponds to the precision of the fixed-point data type (1, 18, 10) used by the LLRF controller.

## 7. Conclusions

In the context of the linear accelerator ELBE, the benefit of implementing a beam-based feedback regulator using an FPGA is twofold. Firstly, the low-latency nature of FPGAs allows dealing with fast processes, such as the regulation of an electron bunch arrival time. Secondly, the high configurability of FPGAs enables the implementation of sophisticated regulation algorithms, e.g., an optimal $H_{2}$ regulator in its state-space representation.

Accordingly, in this work we proposed an FPGA-based implementation of a beam-based feedback regulation algorithm to compensate electron bunch arrival time fluctuations. Using a top-down approach, we gradually introduced the levels of the regulator system, starting from the structure of the ELBE beam-based feedback loop and culminating in the digital architecture of a state-space regulator. In order to implement the matrix-vector multiplications of the state-space formalism, we used systolic arrays. Even though the systolic arrays added data management overhead into the design, we saw that the design could easily be scaled in terms of the regulator orders as well as its inputs and outputs. Furthermore, we made a thorough analysis of a potential fixed-point implementation and then compared it to a floating-point one. Essentially, when clocked at 240 MHz the fixed-point implementation of a 4th-order $H_{2}$ regulator takes 0.425 μs to perform its state-space computations, thus enabling sub-microsecond sample times. In contrast, a similar floating-point implementation takes 1.025 μs. Finally, we verified the correctness of the implementation by running a hardware testbench which included two interconnected FPGAs.

The next step is to validate the proposed digital solution on the real machine. This will allow evaluating the whole spectrum of decisions made in this and our previous works, including the disturbance modeling, the choice of the regulation algorithm and the state-space implementation. Such evaluation will be the subject of our future report.

## Figures and Tables

**Table 1:** Gain matrices with value magnitude ranges and assigned fixed-point data types.

Gain	Minimum Magnitude	Maximum Magnitude	Fixed-Point Data Type
Θ	$-7.3538\times 10^{-5}$	−1.0294	(1, 18, 16)
Ξ	0.0941	−4.1177	(1, 18, 14)
Υ	−0.0012	−0.4782	(1, 18, 16)
Φ	$-3.8253\times 10^{-4}$	0.1224	(1, 18, 16)
Ψ	0.4896	0.4896	(1, 18, 16)
Ω	−0.4896	−0.4896	(1, 18, 16)

**Table 2:** Use of DSP resources by the two main computation units of the state-space algorithm.

Unit	Implementation	DSPs	Clock Cycles	Total DSPs for 7th Order
MAC	fixed-point	1	5	26
	floating-point	4	22	104
Sum	fixed-point	none	3	none
	floating-point	2	14	18

## Data Availability

Not applicable.

## Abbreviations

AMC	Advanced mezzanine card
API	Application programming interface
BAM	Bunch arrival time monitor
BBF	Beam-based feedback
CPU	Central processing unit
CW	Continuous wave
DAQ	Data acquisition
DDR	Double data rate
DSP	Digital signal processor
ELBE	Electron linear accelerator for beams with high brilliance and low emittance
FPGA	Field-programmable gate array
FSM	Finite state machine
HZDR	Helmholtz-Zentrum Dresden-Rossendorf
HW	Hardware
II	Internal interface
IP	Intellectual property
LLL	Low-latency link
LLRF	Low-level radio frequency
MAC	Multiply-accumulate
MTCA	Micro telecommunications computing architecture
PCIe	Peripheral component interconnect express
RAM	Random-access memory
RF	Radio frequency
SFP	Small form-factor pluggable
SSPA	Solid-state power amplifier
SW	Software
UML	Unified modeling language
VHDL	VHSIC hardware description language
VHSIC	Very high-speed integrated circuit
VM	Vector modulator

## Nomenclature

**ELBE accelerator**	
*A*	RF field amplitude
*a*	RF field amplitude change in percents
ϕ	RF field phase
$e^{-}$	electron
τ	electron bunch arrival time in dimensionless BAM modulation units
*I*	in-phase representation of RF field amplitude and phase
*Q*	quadrature representation of RF field amplitude and phase
**Control configuration**	
*A*	system matrix
$B_{2}$	input matrix
$C_{2}$	output matrix
$D_{22}$	feed-forward matrix
*r*	reference input (setpoint)
*e*	error signal
*u*	control signal (manipulated plant input)
*x*	control state
*y*	plant output
$z^{-1}$	discrete integrator
$K_{u}$	state feedback gain
$L_{u}$	observer gain
$L_{x}$	observer gain
Θ	gain from $x\left[k\right]$ to $x\left[k+1\right]$
Υ	gain from $y\left[k\right]$ to $x\left[k+1\right]$
Φ	gain from $u\left[k-1\right]$ to $x\left[k+1\right]$
Ξ	gain from $x\left[k\right]$ to $u\left[k\right]$
Ψ	gain from $y\left[k\right]$ to $u\left[k\right]$
Ω	gain from $u\left[k-1\right]$ to $u\left[k\right]$

## References

[1] Schlarb H. Techniques for Pump-Probe Synchronisation of Fsec Radiation Pulses. Proceedings of the Particle Accelerator Conference.

[2] Pfeiffer S., Branlard J., Hoffmann M., Schmidt C. Advanced LLRF System Setup Tool for RF Field Regulation of SRF Cavities. Proceedings of the 19th International Conference on RF Superconductivity.

[3] Zenker K., Gümüş C., Hierholzer M., Michel P., Pfeiffer S., Schlarb H., Schmidt C., Schurig R., Steinbrück R., Kuntzsch M. (2021). MicroTCA.4-Based Low-Level RF for Continuous Wave Mode Operation at the ELBE Accelerator. IEEE Trans. Nucl. Sci..

[4] Maalberg A., Kuntzsch M., Petlenkov E. Regulation of the Linear Accelerator ELBE Exploiting Continuous Wave Mode of a Superconducting RF Cavity. Proceedings of the American Control Conference.

[5] Kwakernaak H. (2002). *H*_2_-optimization—Theory and applications to robust control design. Annu. Rev. Control.

[6] Skogestad S., Postlethwaite I. (2005). Multivariable Feedback Control: Analysis and Design.

[7] Pfeiffer S., Schmidt C., Bock M.K., Schlarb H., Jalmuzna W., Lichtenberg G., Werner H. Fast Feedback Strategies for Longitudinal Beam Stabilization. Proceedings of the 2012 International Particle Accelerator Conference.

[8] Koprek W., Behrens C., Bock M.K., Felber M., Gessler P., Schlarb H., Schmidt C., Schulz S., Steffen B., Wesch S. Intra-train Longitudinal Feedback for Beam Stabilization at FLASH. Proceedings of the 32nd International Free Electron Laser Conference.

[9] Rezaeizadeh A., Schilcher T., Smith R. MPC based Supervisory Control Design for a Free Electron Laser. Proceedings of the 54th Conference on Decision and Control.

[10] Rezaeizadeh A., Schilcher T., Smith R. (2016). Adaptive robust control of longitudinal and transverse electron beam profiles. Phys. Rev. Accel. Beams.

[11] Bellandi A., Butkowski Ł., Dursun B., Eichler A., Gümüş C., Kuntzsch M., Nawaz A., Pfeiffer S., Schlarb H., Schmidt C. (2021). Online Detuning Computation and Quench Detection for Superconducting Resonators. IEEE Trans. Nucl. Sci..

[12] Löhl F. (2009). Optical Synchronization of a Free-Electron Laser with Femtosecond Precision. Ph.D. Thesis.

[13] Bock M.K. (2013). Measuring the Electron Bunch Timing with Femtosecond Resolution at FLASH. Ph.D. Thesis.

[14] Kuntzsch M., Zenker K., Maalberg A., Schwarz A., Czwalinna M.K., Kral J. Update of the Bunch Arrival Time Monitor at ELBE. Proceedings of the 13th International Particle Accelerator Conference.

[15] Rybaniec R., Przygoda K., Cichalewski W., Ayvazyan V., Branlard J., Butkowski Ł., Pfeiffer S., Schmidt C., Schlarb H., Sekutowicz J. (2017). FPGA-Based RF and Piezocontrollers for SRF Cavities in CW Mode. IEEE Trans. Nucl. Sci..

[16] Wibowo S.B., Matsumoto T., Michizono S., Miura T., Qiu F., Liu N. (2018). Digital low level rf control system for the International Linear Collider. Phys. Rev. Accel. Beams.

[17] Qiu F., Michizono S., Miura T., Matsumoto T., Liu N., Wibowo S.B. (2018). Real-time cavity simulator-based low-level radio-frequency test bench and applications for accelerators. Phys. Rev. Accel. Beams.

[18] St. John J., Herwig C., Kafkes D., Mitrevski J., Pellico W.A., Perdue G.N., Quintero-Parra A., Schupbach B.A., Seiya K., Tran N. (2021). Real-time artificial intelligence for accelerator control: A study at the Fermilab Booster. Phys. Rev. Accel. Beams.

[19] Kumar V.K.P., Tsai Y.-C. Synthesizing optimal family of linear systolic arrays for matrix computations. Proceedings of the International Conference on Systolic Arrays.

[20] Jang J.-W., Choi S.B., Prasanna V.K. (2005). Energy- and time-efficient matrix multiplication on FPGAs. IEEE Trans. Very Large Scale Integr. VLSI Syst..

[21] Shen J., Qiao Y., Huang Y., Wen M., Zhang C. Towards a Multi-array Architecture for Accelerating Large-scale Matrix Multiplication on FPGAs. Proceedings of the 2018 International Symposium on Circuits and Systems.

[22] Wei X., Yu C.H., Zhang P., Chen Y., Wang Y., Hu H., Liang Y., Cong J. Automated Systolic Array Architecture Synthesis for High Throughput CNN Inference on FPGAs. Proceedings of the 54th Annual Design Automation Conference.

[23] Walter T., Ludwig F., Rehlich K., Schlarb H. Novel Crate Standard MTCA.4 for Industry and Research. Proceedings of the 4th International Particle Accelerator Conference.

[24] Kuntzsch M., Steinbrück R., Schurig R., Hierholzer M., Killenberg M., Schmidt C., Hoffmann M., Iatrou C., Rahm J., Rutkowski I. MicroTCA.4-Based LLRF for CW Operation at ELBE - Status and Outlook. Proceedings of the 6th International Beam Instrumentation Conference.

[25] Butkowski Ł., Kozak T., Prędki P., Rybaniec R., Yang B.Y. FPGA Firmware Framework for MTCA.4 AMC Modules. Proceedings of the 15th International Conference on Accelerator and Large Experimental Physics Control Systems.

[26] Data Processing AMC Module for MTCA & ATCA. https://www.nateurope.com/products/NAT-AMC-TCK7.html.

[27] Killenberg M., Petrosyan L.M., Schmidt C., Marsching S., Piotrowski A. Drivers and Software for MTCA.4. Proceedings of the 5th International Particle Accelerator Conference.

[28] Dimitrakopoulos G., Psarras A., Seitanidis I. (2015). Microarchitecture of Network-on-Chip Routers: A Designer’s Perspective.

[29] Clements A. (2006). Principles of Computer Hardware.

[30] 7 Series FPGAs Data Sheet: Overview. https://docs.xilinx.com/v/u/en-US/ds180_7Series_Overview.

[31] 7 Series DSP48E1 Slice: User Guide. https://docs.xilinx.com/v/u/en-US/ug479_7Series_DSP48E1.

[32] (2019). IEEE Standard for Floating-Point Arithmetic. (Revision of IEEE 754-2008).

[33] Floating-Point Operator v7.1: LogiCORE IP Product Guide. https://docs.xilinx.com/v/u/en-US/pg060-floating-point.

